# Embolization of middle meningeal artery (EMMA) for non-acute subdural hematoma: Insight from recent randomized trials and meta-analysis

**DOI:** 10.1177/15910199251318408

**Published:** 2025-02-03

**Authors:** Jai Shankar

**Affiliations:** 1 Department of Radiology, University of Manitoba, Winnipeg, Manitoba, Canada

**Keywords:** Embolization of middle meningeal artery, non-acute subdural hematoma, middle meningeal artery, metanalysis

## Abstract

Embolization of the middle meningeal artery (EMMA) has emerged as a promising treatment for non-acute subdural hematoma (NASDH), either as an adjunct to surgical drainage or as a primary intervention in patients not undergoing surgery. Recent randomized controlled trials (RCTs) have investigated the efficacy of EMMA using dimethyl sulfoxide (DMSO)-based agents like ONYX and SQUID. The EMBOLISE trial demonstrated a significant reduction in hematoma recurrence with adjunctive EMMA, while the STEM trial showed similar benefits at 180 days. Conversely, the MAGIC MT trial found no significant difference in recurrence rates with EMMA. A meta-analysis of these trials confirmed EMMA's safety, with no significant increase in serious adverse events. The analysis indicated a modest overall benefit in reducing NASDH recurrence (risk difference −0.09, *P* = 0.02), though results were largely driven by the STEM trial. The benefit of adjunctive EMMA was less clear, with no significant effect found. Primary EMMA showed marginal benefit but with considerable variability. Factors such as primary outcome, trial design, patient demographics, and surgical biases complicate the interpretation of these findings. While the safety of EMMA is supported, its clinical efficacy remains inconclusive. Further trials, including patient-level meta-analyses, are needed to refine the role of EMMA in NASDH management and address existing gaps in the literature.

Embolization of middle meningeal artery (EMMA) has emerged as a potential treatment for non-acute subdural hematoma (NASDH), either as an adjunctive therapy following surgical drainage or as a primary intervention for patients who do not undergo surgery.^1–3^ The primary goal of EMMA is to reduce the risk of NASDH recurrence after surgical drainage and offer a treatment option for patients at high surgical risk. Although earlier studies have generally been small or involved meta-analyses, randomized controlled trials (RCTs) are crucial for determining the widespread applicability of EMMA in NASDH management.

Recent RCTs have assessed the efficacy of EMMA in treating NASDH, using dimethyl sulfoxide (DMSO)-based embolic agents like ONYX (Medtronic Inc) and SQUID (Balt Inc) either as a primary treatment or an adjunct to surgical drainage.^4–6^ These trials provide the first level one evidence supporting EMMA for NASDH.

In the EMBOLISE trial, adjunctive EMMA following surgical drainage led to a significant reduction in hematoma recurrence or progression requiring repeat surgery at 180 days (4.1% in the treatment group vs. 11.3% in the control group; relative risk, 0.36; 95% confidence interval [CI], 0.11–0.80; *P* = 0.008).^6^ Both MAGIC MT and STEM included EMMA as primary treatment or adjunct to surgical drainage. MAGIC MT did not find a significant difference in recurrence rates at 90 days (6.7% in the EMMA group and 9.9% in the control group; risk difference, −3.3; 95% CI, −7.4 to 0.8; *P* = 0.10).^5^ The STEM trial demonstrated a significant reduction in the composite primary efficacy outcome at 180 days (16% in the EMMA group and in 36% in the control group; odds ratio, 0.36; 95% CI, 0.20–0.66; *P* = 0.001).^4^ The EMBOLISE and STEM trials suggest that EMMA may reduce the risk of recurrence or progression of NASDH compared to standard care. However, the MAGIC MT trial showed neutral results, indicating no significant benefit of EMMA in preventing recurrence. These inconsistent findings highlight the need for further evaluation and clarification of EMMA's role in NASDH management.^7^

A meta-analysis was performed based on the published data from these three trials using RevMan 5.4.1 software. Risk differences were calculated for clinically relevant variables using random effect. For this the pre-specified primary outcome was used for each trial.

While the efficacy varied across the studies, the meta-analysis showed that EMMA did not significantly increase the risk of serious adverse events (SAEs), with a risk difference of −0.03 (95% CI, −0.08 to 0.01; *P* = 0.14), confirming the safety of EMMA.

For efficacy analysis, considering the predetermined primary outcome of each study, the meta-analysis indicated an overall benefit of EMMA in reducing NASDH recurrence (risk difference −0.09, 95% CI, −0.17 to −0.01; *P* = 0.02). (Figure 1(a)). The number needed to treat of 11. However, this effect size is largely driven by that of STEM trial.

**Figure 1.**
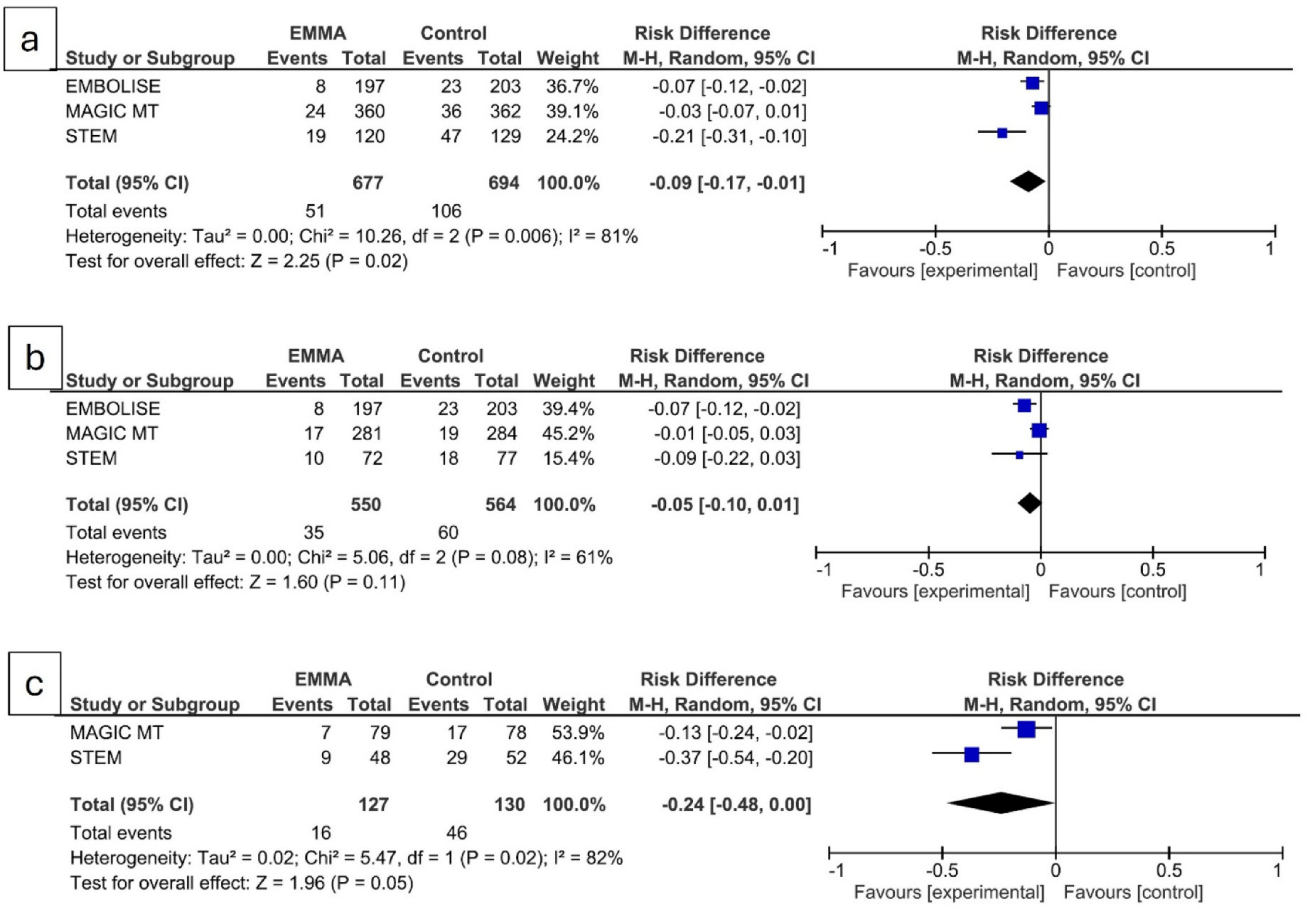
(a) Forest plot comparing the overall risk difference in the primary outcome between embolization of the middle meningeal artery (EMMA) and control in patients with non-acute subdural hematoma. (b) Forest plot comparing the risk difference for EMMA adjunctive to surgical drainage. (c) Forest plot comparing the risk difference for primary EMMA.

Notably, the effect of adjunctive EMMA versus primary EMMA should be interpreted separately as EMMA is a second intervention when done adjunctive to surgical drainage.^8^ In EMBOLISE, all patients received adjunctive EMMA, while in MAGIC MT and STEM, fewer patients received this treatment (78.25% and 59.84%, respectively). Only EMBOLISE showed benefit of adjunctive EMMA and both MAGIC MT and STEM did not show any benefit. The meta-analysis showed no significant benefit of adjunctive EMMA with a risk difference of −0.05 (95% CI, −0.010 to 0.01; P = 0.11) (Figure 1(b)).

None of the patients underwent primary EMMA in EMBOLISE trial and only smaller subset underwent primary EMMA in MAGIC MT and STEM trials (21.75% and 40.16%, respectively). Primary EMMA showed benefit in both these trials with variable effect size. On meta-analysis, primary EMMA showed a marginal benefit (risk difference - 0.24 (95% CI, −0.048 to −0.00; P = 0.05) (Figure 1(c)). The wide CI reflects smaller number of patients in this subgroup.

The ongoing debate within the neurointerventional community concerns the implementation of EMMA in clinical practice. While the evidence from the meta-analysis suggests some benefit of EMMA, the heterogeneous patient populations and differing primary outcomes across trials complicate its generalization. Regulatory bodies must also consider the higher cost associated with EMMA, which is nearly double that of standard care (44,284 ± 23,310 for EMMA vs. 21,490 ± 16,632 for standard care).^5^

A limitation of this meta-analysis is that it was based solely on published data. A more detailed, patient-level meta-analysis could provide deeper insights and help answer several important questions.

Sources of biases in these trials- All three trials allowed the use of EMMA prior to surgical drainage. Specifically, 66%, 99.6%, and 100% of patients in the EMBOLISE, MAGIC MT, and STEM trials, respectively, received EMMA before surgical intervention. However, the lack of blinding in the surgical decision-making process introduces a potential risk of bias. Neurosurgeons might alter their approach based on their knowledge of a patient's treatment group, which could influence the outcomes. This is particularly concerning given that the primary outcome, which involved repeat or rescue surgery, was assessed by unblinded operators, further increasing the risk of bias. The decision to perform repeat or rescue surgery can be highly subjective and may vary significantly between neurosurgeons, especially for patients who have not undergone prior surgical drainage. Presence of radio-opaque embolic material used for EMMA on the follow-up CT scans is another challenge for blinding. However like many trials with implantable device and imaging endpoint, blinding is not possible on the follow-up in EMMA patients.

Furthermore, the inclusion of patients with bilateral NASDH in some trials introduces another potential bias. In fact, approximately one-fifth of patients in EMBOLISE, MAGIC MT, and STEM trials (19.75%, 20.9%, 20%, respectively) had bilateral NASDH. Bilaterality of NASDH could confound the assessment of any unilateral focal neurological deficits either at the time of enrolment or at the time of follow-ups.

Additionally using non-resolution of hematoma as a primary outcome raises concerns about the relevance, particularly for older patients with NASDH.^4^ However, only 12% (8 out of the 66) of the primary outcomes were classified as recurrent or residual hematoma, and the exact number of patients with residual hematoma was not reported.

Furthermore, the difference in patient populations should not be overlooked. The trial conducted in a Chinese cohort did not demonstrate a benefit of EMMA in NASDH patients. In contrast, trials involving more diverse patient populations—though predominantly white—showed a positive effect of EMMA in treating NASDH. These variations in patient demographics highlight the need for further investigation into how different populations may respond to EMMA treatment.

Loss to follow-up in trials such as EMBOLISE (13.2%) and STEM (15%) also complicates the interpretation of results, especially when the treatment effect is small. Although similar outcome without imputation of missing data is reassuring in both of these trials, these factors underscore the need for more robust and well-designed studies to assess the true efficacy and safety of EMMA.

In conclusion, while recent trials have demonstrated the safety of EMMA for NASDH, our meta-analysis suggests that the evidence regarding its efficacy—particularly as an adjunct to surgical drainage—remains inconclusive. The results from ongoing trials are eagerly awaited, as they may offer clearer insights and address the current gaps in understanding. A further detailed meta-analysis of all trials with patient-level data is also warranted and could provide further insight. With careful consideration of trial design and patient selection, EMMA may play a more defined role in the management of NASDH in the future.
